# Surgical Outcomes in Rare Movement Disorders: A Report of Seventeen Patients from India and Review of Literature

**DOI:** 10.5334/tohm.693

**Published:** 2022-06-20

**Authors:** Debjyoti Dhar, Vikram Venkappayya Holla, Nitish Kamble, Ravi Yadav, Dwarakanath Srinivas, Pramod Kumar Pal

**Affiliations:** 1 Department of Neurology, National institute of Mental Health and Neurosciences (NIMHANS, India – 560029), IN; 2 Neurosurgery, National institute of Mental Health and Neurosciences (NIMHANS, India – 560029), IN

**Keywords:** Functional neurosurgery, pallidotomy, thalamotomy, DBS, Rare movement disorders

## Abstract

**Background:**

Rare movement disorders (RMDs) throw remarkable challenges to their appropriate management particularly when they are medically refractory. We studied the outcome of functional neurosurgery among patients with RMDs.

**Methods:**

Retrospective chart-review from 2006 to 2021 of patients with RMDs who underwent either Deep brain Stimulation (DBS) or lesional surgeries in the department of Neurology and Neurosurgery at a tertiary care centre.

**Results:**

Seventeen patients were included. Generalized dystonia (11 patients, 64.7%) and tremor (5 patients, 29.4%) were the most common indication for surgery whereas, Wilson’s disease (8 patients, 47.1%) and Neurodegeneration with brain iron accumulation (5 patients, 29.4%) were the most common aetiology. Sixteen patients (94.1%) had objective clinical improvement. Significant improvement was noted in the dystonia motor scores both at 6-months and 12-months follow-up (n = 11, p-value of <0.01 and 0.01 respectively). Comparison between DBS and lesional surgery showed no significant difference in the outcomes (p = 0.95 at 6-months and p = 0.53 at 12-months), with slight worsening of scores in the DBS arm at 12-months. Among five patients of refractory tremor with Wilson’s disease, there was remarkable improvement in the tremor scores by 85.0 ± 7.8% at the last follow-up. Speech impairment was the main complication observed with most of the other adverse events either transient or reversible.

**Discussion:**

Surgical options should be contemplated among patients with disabling medically refractory RMDs irrespective of the aetiology. Key to success lies in appropriate patient selection. In situations when DBS is not feasible, lesional surgeries can offer an excellent alternative with comparable efficacy and safety.

## Introduction

Rare diseases (RD) are defined as those with a prevalence of less 200,000 people in United States [1]. On the contrary, the European Union under the jurisdiction of the European Medical Agency considers RDs as prevalence of less than or equal to 50 people per 100,000 population. As of now, 6000 to 8000 rare diseases have been discovered so far which has been estimated to affect 6–8% of the global population [1]. Collectively, they add up to significant burden of disease in the overall population. RDs pose remarkable challenges to the formulation of management strategies. Many of them are life-threatening or disabling which raises ethical concerns with regards to the conduct of controlled or randomised clinical trials. Thus, in most of these situations, treatment decisions are guided by evidence-limited medicine and principles of good clinical practice.

As per European Reference Network (ERN) for rare neurologic disorders (RND), neurologic manifestations are widely prevalent among RDs [2]. Movement disorders constitute one of the most important phenotypes in rare neurological disorders [2], treatment of which is often challenging with definite medical therapy available in only a handful of conditions. Often, the associated movement disorder responds unsatisfactorily to the standard medical management [3]. Since the definitive therapies for majority of the RMDs are still lacking, symptomatic management by means of surgical interventions in these often disabling and intractable movement disorders can play an immense role in improving the quality of living. With the refinement of surgical techniques, the past few decades have witnessed a significant upsurge in the role of surgical interventions as a part of treatment protocol in medically refractory movement disorders of various aetiologies. The evolution of Deep Brain Stimulation (DBS) technology has led to a decline in the number of publications related to ablative surgeries in recent times because of several advantages in the former such as reversibility, programmability, and bilateral targeting ability. Studies have also shown persistent beneficial effects of bilateral GPi DBS in patients with dystonia in long-term follow-up [4,5]. Younger age at surgery, higher BFMDRS motor and disability scores were associated with better clinical response to DBS [4]. GPi stimulation were associated with a greater clinical benefit compared to posterior ventro-lateral (VLp) thalamic stimulation [6]. The reversibility of DBS and its modulatory effects on neural plasticity in patients with dystonia after prolonged stimulation has been robustly demonstrated by means of transcranial magnetic stimulation [7]. Experience of DBS in Parkinson’s disease has shown the large economic burden of DBS. However, in the long run, it successfully reduces the financial burden of pharmacotherapy and indirectly the social costs bu improving the quality of life [8]. Robust cost-effectiveness studies of DBS in movement disorders are still lacking, and remains an area of further research [9]. There has been significant improvement of health-related quality of life (HRQoL) in patients with dystonia post-DBS, particularly with primary dystonia [10]. Nevertheless, lesional surgeries still constitute a very crucial part of the armamentarium in the management [11,12]. This modality of management is particularly important in patients with financial constraints, poor compliance to follow-up visits and those at prone for infections.

Outcomes of surgical interventions in RMDs are less well-known in the literature. There is a dearth of studies on the impact of lesional surgeries and DBS among patients with RMDs. We aimed to discuss our experience over a period of past 15 years on the various surgical interventions performed among patients with RMDs with drug refractory dystonia, tremors, chorea and tics.

## Methods

### Subject recruitment

We performed a retrospective review of all the patients with rare movement disorders who underwent DBS or lesioning surgeries from 2006 to 2021 in our institute. A detailed review of all the charts was done. Data were extracted with respect to the diagnoses, duration of symptoms, indications for surgery, medical management tried, the type of surgery performed, pre-operative and post-operative objective assessment and the subsequent long-term outcome. Analyses were performed on the measured clinical scores which included Burke-Fahn-Marsden-Dystonia-Rating Scale (BFMDRS) for dystonia, Fahn-Tolosa-Marin (FTM) scores for tremors, Unified Huntington Disease Rating Scale (UHDRS) for chorea, Yale Global Tic Severity Scale (YGTSS) for tics, Yale-Brown obsessive compulsive severity scale (YBOCSS)for obsessive compulsive symptoms, Hamilton depression rating scale (HDRS) for depression, and Hamilton anxiety rating scale (HARS) for anxiety. Five previously published patients by our centre, three patients of Wilson’s disease (WD), one patient of Neurodegeneration with brain iron accumulation (NBIA) and one patient of Tourette’s syndrome, were also included in the study [13,14,15]. The Institute Ethics Committee at the National Institute of Mental Health and Neurosciences granted an ethical clearance waiver owing to the retrospective nature of the study with de-identified data being extracted from files, and informed written consent obtained from patients for publication of recorded videos.

### Statistical analysis

Quantitative variables were expressed as frequency and mean ± S.D. Descriptive analyses were done to obtain the demographic parameters and clinical profile. Repeated-measures ANOVA was used to compare the BFMDRS scores between the independent groups comprising lesioning and DBS as well as overall study population, at baseline, 6-months and 12-months post-surgery with post-hoc analysis wherever applicable. Independent samples T-test was applied between the two groups, lesional surgery and DBS, at each of the time frames. Surgical outcomes were also compared between the two disease groups, WD and NBIA at 6-months and 12-months post-surgical intervention. These tests were corrected using Bonferroni correction. Statistical analyses were applied only on patients with dystonia. A p-value of less than 0.05 was taken as statistically significant. Statistics were performed using IBM SPSS version 23. Graph-pad Prism 8 was used for synthesis of figures.

## Results

**Figure 1:**
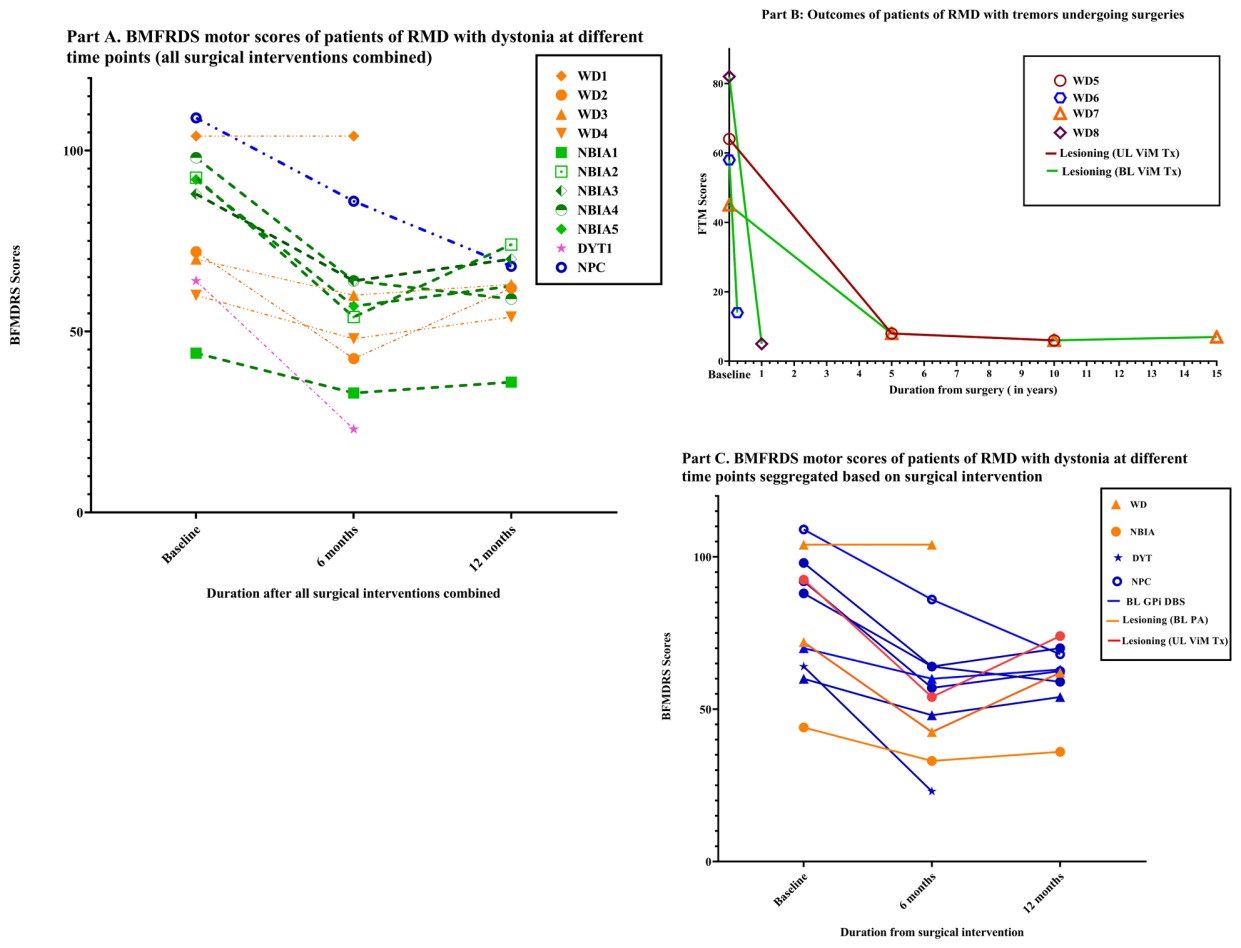
BMFDRS motor scores at baseline, 6-months and 12-months post operatively in patients with dystonia.

Seventeen patients (11 males, 64.7%) were included in the study (Table 1). The mean age at onset was 13.7 ± 8.9 years with the mean duration of illness prior to the surgery of 5.9 ± 3.7 years. The mean age at the time of the surgery was 19.8 ± 9.7 years, with a range of 6 years to 32 years. The most common underlying aetiology was WD (8 patients, 47.1%) followed by NBIA (5 patients, 29.4%), and the predominant phenomenology at the time of surgeries was dystonia in 11 patients (64.7%) and tremor in 5 patients (29.4%). Among patients with dystonia, 6-month follow-up data of BFMDRS motor severity scores were available in all and 12-month follow-up scores were available for 9 patients. Lack of data of these two patients was attributable to the unfortunate demise of one patient of WD from hepatic failure and the absence of follow-up of the patient with DYT-TOR1A (Table 1, Figure 1). Genetic testing was performed in 3 cases. Patient DYT1 had previously reported heterozygous 3-bp deletion (c.907_909del) in exon-5 of *TOR1A* gene, patient NPC had novel homozygous likely pathogenic missense variant (c.2473T > C;p.Tr825His) in exon-16 of *NPC1* gene, and patient NAC had novel homozygous pathogenic nonsense variant (c.9477G < > A;p.Trp3159Ter) in exon-72 of *VPS13A* gene. Clinical vignette is provided for patient NBIA4, WD8, DYT1, and NBIA2 along with videos for patient NBIA4 (Video e1), WD8 (Video e2), and DYT1 (Video e3).

**Table 1:** Demographics, clinical profile, surgical interventions and comparison between DBS and lesioning surgery.


VARIABLES(N,% OR MEAN ± S.D)	TOTAL	LESIONING	DBS	REMARKS

Number of patients	17	8	9	

Mean age at onset (y)	13.7 ± 8.9	13.5 ± 7.2	18.8 ± 10.9	

Mean age at surgery (y)	19.8 ± 9.7	21.0 ± 8.7	19.2 ± 11.6	

Mean duration of illness before surgery (y)	5.9 ± 3.7	6.5 ± 5.2	4.8 ± 1.8	

Underlying diagnosis				

Wilson’s disease	8 (47.1%)	6 (75%)	2 (22.2%)	

NBIA spectrum disorder	5 (29.4%)	2 (25%)	3 (33.3%)	

DYT-TOR1A	1 (5.9%)	0	1 (11.1%)	

Neuroacanthocytosis	1 (5.9%)	0	1 (11.1%)	

Niemann-Pick disease type C	1 (5.9%)	0	1 (11.1%)	

Giles de la Tourette syndrome	1 (5.9%)	0	1 (11.1%)	

Indication for surgery^@^				

Generalized dystonia	11 (64.7%)	4(50%)	7 (77.8%)	

Tremors	5 (29.4%)	5(62.5%)	0	

Generalized chorea	1 (5.9%)	0	1 (11.1%)	

Complex motor tics	1 (5.9%)	0	1 (11.1%)	

Type surgery				

Unilateral ViM thalamotomy	4 (23.5%)	4 (50%)	NA	

		(Rt-1, Lt-3)		

Bilateral ViM thalamotomy	1 (5.9%)	1 (12.5%)	NA	

Bilateral pallidotomy	3 (17.7%)	3 (37.5%)	NA	

Bilateral GPi DBS	9 (52.9%)	NA	9 (100%)	

Complications				

Speech impairment	7 (41.2%)	4 (44.4%)	1 (12.5%)	

Swallowing difficulty	3 (17.7%)	3 (33.3%)	0	

Movement disorder				

– Blepharospasm	3 (17.7%)	1 (11.1%)	2 (25.0%)	

– Status dystonicus	1 (5.9%)	1 (11.1%)	0	

– Persistent dystonia	5 (29.4%)	2 (22.2%)	3 (37.5%)	

– Persistent choreiform movements	1 (5.9%)	1 (11.1%)	0	

– Persistent tremor	1 (5.9%)	0	1 (12.5%)	

– Persistent tongue dystonia	2 (11.8%)	0	2 (25.0%)	

– Perioral tightness	1 (5.9%)	1 (11.1%)	0	

– Bradykinesia	2 (11.8%)	1 (11.1%)	1 (12.5%)	

– Parkinsonism	1 (5.9%)	1 (11.1%)	0	

Gait impairment	2 (11.8%)	1 (11.1%)	0	

Surgical Site infection	1 (5.9%)	1 (11.1%)	0	

*Dystonia subgroup*	11	4	7	

– Pre-operative baseline BFMDRS score	80.9 ± 20.0	78.1 ± 26.3	82.4 ± 17.6	p value 0.75

– BFMDRS score at 6 months post-surgery	57.8 ± 22.8	58.3 ± 31.6	57.4 ± 19.1	p value 0.95

– Percentage reduction of BFDRS score at 6 m	29.6 ± 16.6	26.9 ± 19.5 %	31.6 ± 16.2 %	

– BFMDRS mean score difference at baseline and 6 m	p value < 0.001	p value 0.11	p value 0.001	

– BFMDRS score at 12 months post-surgery	60.9 ± 11.1	57.3 ± 11.2 (n = 3)	62.7 ± 5.8 (n = 6)	p value 0.53

– Percentage reduction of BFDRS score at 12 m compared to baseline	22.3 ± 10.4	17.4 ± 3.1 %	24.8 ± 12.1 %	

– BFMDRS mean score difference at baseline and 12 m	p value 0.02	p value 0.06	p value 0.010	

– BFMDRS mean score difference between 6 m and 12 m	p value 0.28	p value 0.13	p value 0.92	


@-In one patient, the indication of surgery was tremor and dystonia. BFMDRS: Burke-Fahn-Marsden-Dystonia-Rating scale, DBS-deep brain stimulation, GPi-globus pallidus interna, Lt-left, m-month, NA-not applicable, Rt-right, ViM-ventralis intermediate nucleus, y-years.

**Video e1:** **Video of the patient-NBIA4.** Segment-1: pre-operative video showing severe generalized dystonia with opisthotonus and Ryle’s tube in situ due to dysphagia. Segment-2: Video of the patient 6-months after bilateral GPi-DBS showing improvement in dystonia, opisthotonus and dysphagia. The video was taken after written informed consent for online publication and dissemination.

**Video e2:** **Video of the patient-WD8.** *Segment-1*: pre-operative video showing significant disabling rubral tremor in right upper limb with conducted tremor in left upper limb as well. *Segment-2*: 6-months after left ViM thalamotomy showing significant improvement of the tremor and the functional ability. The video was taken after written informed consent for online publication and dissemination.

**Video e3:** **Video of the patient-DYT1.** *Segment-1*: pre-operative video showing significant generalized dystonia. *Segment-2*: 3-months post bilateral GPi-DBS with significant improvement in generalized dystonia as well as gait. The video was taken after written informed consent for online publication and dissemination.

### Treatment prior to the surgery

The patients were tried on symptomatic medical therapy which included clonazepam, baclofen, tetrabenazine, trihexyphenidyl and decoppering agents for a mean duration of 4.6 ± 2.6 years. In patients with WD, the mean daily dose of D-Penicillamine was 937.5 ± 320.0 mg while for Zinc, it was 632.5 ± 341.6 mg. Among patients with primary and secondary dystonia, the mean daily dosage of trihexyphenidyl, tetrabenazine, baclofen, clonazepam and diazepam were 13.6 ± 5.3 mg, 87.5 ± 19.8 mg, 26.8 ± 13.1 mg, 1.5 ± 0.9 mg and 8.8 ± 1.8 mg respectively. Patients with severely disabling movement disorders and suboptimal response to the maximal therapeutic and tolerated dosage of the drugs were considered for the surgical intervention after a detailed discussion with patient and the caregivers.

### Surgical intervention: Deep Brain Stimulation

Nine patients (52.9%) underwent DBS with the mean duration of illness prior to DBS of 4.8 ± 1.8 years (Tables 1, 2). Seven patients (77.8%) underwent DBS for dystonia with underlying aetiology of NBIA spectrum disorders in three patients (33.3%), WD in two patients (22.2%), and Niemann-Pick Type C (NPC) and DYT-TOR1A in one patient each (11.1%). Other indications were chorea due to Neuroacanthocytosis in one patient and complex motor and vocal tics in Tourette syndrome in one patient. All these patients underwent bilateral Globus Pallidus interna (GPi) DBS.

**Table 2:** Individual patient characteristics.


ID	INDICATION	AAO (YRS)	AAP (YRS)	AAS (YRS)	DOI (YRS)	SEX	MEDICAL MANAGEMENT	SX TYPE	OUTCOME	COMPLICATIONS	STIMULATION PARAMETERS	MANAGEMENT IN FOLLOW UP	OUTCOME

WD1	Dystonia	8	8	11	3	F	Penicillamine 1 gm THP 6 mg Zinc sulfate 220 mg Baclofen 20 mg	BL GPi Px	No improvement (BFMDRS scores 104, which remained unchanged at 6 months)	Expired from hepatic failure	NA	-----	-----

WD2	Dystonia	15.5	16	17	1.5	M	Penicillamine 1 gm THP 18 mg Zinc sulfate 660 mg TBZ 100 mg CBZ 400 mg Baclofen 20 mg	BL GPi Px	Improvement of BFMDRS scores by 40.9% at 6 months and 13.9% at 12 months	Persistent disabling dystonia, blepharospasm, tongue dystonia	NA	Redo bilateral pallidotomy at 14 months post-surgery. Medical management with Penicillamine 1 gm, THP 18 mg, Zinc sulphate 660 mg, TBZ 75 mg, Baclofen 10 mg and CBZ 400 mg	Blepharospasm improved; Dystonia- not improved

WD3	Dystonia	11.5	12	15	3.5	M	Penicillamine 1.25 gm THP 18 mg Zinc sulfate 220 mg Baclofen 20 mg Clonazepam 1 mg Levodopa 250 mg	BL GPi DBS	Improvement of BFMDRS scores by 16.6 % at 6 months and 12.5% at 12 months.	Persistent swallowing difficulty at 1 month. Perioral tightness	At 2 months C+1-/2.1 V/150 µS/180 Hz C+9/2.1 V/150 µS/180 Hz	Reprogramming Penicillamine 1.25 gm THP 18 mg Zinc sulfate 220 mg Baclofen 20 mg Clonazepam 1 mg Levodopa 250 mg	Perioral tightness improved. dystonia persisted of lesser severity

WD4	Dystonia	11	12	15	4	M	Penicillamine 1 gm Zinc sulfate 660 mg THP 12 mg TBZ 75 mg Levodopa 300 mg Baclofen 20 mg Diazepam 7.5 mg	BL GPi DBS	Improvement of BFMDRS scores by 20% at 6 months and 10% at 12 months	Dystonic spasms	At 3 months C+2-/2 V/150 µS/180 Hz C+10/2 V/150 µS/180 Hz	At 1 year follow up, reprogramming to C+2-/3 V/150 µS/180 Hz C+10-11/2 V/150 µS/180 Hz Penicillamine 1 gm Zinc sulfate 660 mg THP 12 mg TBZ 75 mg Levodopa 300 mg Baclofen 15 mg Diazepam 5 mg	Improvement in frequency of dystonic spasms, dystonia persistent

WD5	Tremor	15	17	23	8	M	Penicillamine 1.25 gm THP 18 mg Zinc sulfate 660 mg Baclofen 20 mg	BL ViM Tx	Tremor scores (FTMRS) improved by 87.5% at 5 years and 90.6% at last follow up (10 years) compared to baseline	Dysarthria, postural tremors, bradykinesia persisted. Worsening of tremors to baseline scores	NA	Bilateral redo thalamotomy after 6 months At 1 year follow up, Penicillamine 1 gm THP 12 mg Zinc sulfate 660 mg Baclofen 20 mg	Sustained improvement with mild dysarthria and tremors. Functionally independent

WD6	Tremor	11	8^#^	28	17	F	Zinc 1320 mg Penicillamine 250 mg Pimozide 250 mg	UL ViM Tx	Tremor scores (FTMRS) improved by 75.9% at 2 months	Mild gait ataxia	NA	At 2 years follow up, Amantadine 100 mg Zinc 660 mg Penicillamine 250 mg	Mild ataxia persisted. Tremors improved.

WD7	Tremor	27	30	30	3	M	Penicillamine 750 mg Zinc sulfate 660 mg THP 6 mg TBZ 75 mg Pimozide 250 mg	UL ViM Tx	FTMRS improved by 82.2% at 5 years and 86.7% at 10 years and 84.4% at 16 years follow-up compared to baseline	Transient gait imbalance, post-operative transient pneumocephalus	NA	At 16 years follow up, Penicillamine 750 mg THP 12 mg Zinc sulphate 660 mg	Tremors improved satisfactorily. Mild dysarthria has persisted

WD8	Tremor	16	20	22	6	F	Penicillamine 1 gm Zinc sulfate 660 mg	UL ViM Tx	FTMRS reduction by 94% at 1 year follow-up	No adverse events reported	NA	At 1 year follow up, Penicillamine 500 mg Zinc sulfate 660 mg Clonazepam 0.75 mg	Significant improvement in tremors

NBIA1	Dystonia	3.5	5	7	3.5	F	TBZ 75 mg Baclofen 30 mg THP 18 mg	BL GPi Px	Improvement of BFMDRS scores by 25% at 6 months and 18.2% at 12 months	Abnormal tongue protrusions	NA	At 1 year follow up, TBZ 37.5 mg, THP 18 mg, Clonazepam 0.5 mg, Baclofen 30 mg and Haloperidol 5 mg and Inj Botulinum toxin therapy in the tongue	Improvement in severity of dystonia

NBIA2	Tremor+ Dystonia	13	30	30	17	M	Baclofen 30 mg THP 18 mg	UL ViM Tx	Improvement of BFMDRS scores by 41.6% at 6 months and 20% at 12 months. FTMRS improvement by 77.8 % at 3 years follow-up.	Febrile illness due to chest infection. Worsening of dystonia after stopping haloperidol	NA	At 3 years follow up, Botox for lingual dystonia Baclofen 20 mg THP 12 mg	Required ICU care and tracheostomy. Dystonia persisted with significant disability. Tremors improved.

NBIA3	Dystonia	7	9	12	5	M	Baclofen 60 mg Levodopa 400 mg TBZ 112.5 mg THP 12 mg Diazepam 10 mg	BL GPi DBS	Improvement of BFMDRS scores by 27.3% at 6 months and 20.5% at 12 months	Improvement in dystonia score	At 3 months C+1- /1.0 V/150 µsec/130 Hz C+9-/1.0 V/150 µsec/130 Hz	At 2 years follow up, reprogramming C+1-/2.0 V/150 µsec/130 Hz C+9-/1.0 V/150 µsec/130 Hz Baclofen 30 mg Levodopa 400 mg TBZ 75 mg THP 12 mg	Persistent dystonia of reduced severity

NBIA4	Dystonia	3	4	6	3	F	THP 19.5 mg Clonazepam 0.75 mg Pimozide 4 mg TBZ 75 mg Baclofen 15 mg	BL GPi DBS	Improvement of BFMDRS scores by 30.4% at 6 months and 35.9% at 12 months	Surgical site infection of cranial and abdominal wound (MRSA). Persistence of morning and nocturnal dystonic spasms	At 3 months, C+1-/3 V/120 µS/160 Hz C+9-/3 V/120 µS/160 Hz	Removal of leads	Improvement of dystonia for next 2 years, re-implantation of DBS leads for worsened dystonia, later expired from chest infection

NBIA5	Dystonia	26	28	32	6	M	Baclofen 20 mg TBZ 75 mg Clonazepam 1 mg Levodopa 300 mg	BL GPi DBS	Improvement of BFMDRS scores by 38.0% at 6 months and 32.1% at 12 months	Blepharospasm, Parkinsonism	At 3 months C+1-/2.5 V/210 µS/60 Hz C+5-/2.5 V/210 µS/60 Hz	At 2 years follow up, reprogramming to C+1-/3.5 V/210 µS//130 Hz C+5-/3.4 V/210 µS/130 Hz,, Inj. Botulinum Toxin and medical management with Baclofen 20 mg TBZ 75 mg Clonazepam 0.75 mg, Levodopa 300 mg	Blepharospasm improved, dystonia persistent of reduced severity

NPC	Dystonia	11	14	20	9	F	THP 12 mg Baclofen 40 mg TBZ 100 mg Clonazepam 1.5 mg	BL GPi DBS	Improvement of BFMDRS scores by 21.1% at 6 months and 37.6% at 12 months	Dystonia persistent, speech abnormality, swallowing difficulty	At 3 months, C+1-/1.5 V/90 µS/180 Hz C+9-/1.5 V/90 µS/180 Hz	At 2 years follow up, reprogramming to C+1-/3.5 V/120 µS/180 Hz C+9-/3.5 V/120 µS/180 Hz THP 12 mg Baclofen 40 mg TBZ 50 mg Clonazepam 1.5 mg	Dystonia severity reduced but persistent

DYT	Dystonia	9	11	13	4	M	THP 12 mg TBZ 75 mg Levodopa 400 mg	BL GPi DBS	Improvement of BFMDRS scores by 64.1% at 6 months	Persistence of dystonia	At 3 months C+1-/1.3 V/150 µS/150 Hz C+9-/1.3 V/150 µS/150 Hz	At 9 months follow up, THP 12 mg TBZ, Levodopa tapered and stopped	Improvement in severity of dystonia

NAC	Chorea	37	38	41	4	M	TBZ 125 mg CBZ 600 mg Clonazepam 0.5 mg Baclofen 20 mg	BL GPi DBS	Improvement in UHDRS scores by 9.1 % at 6 months and 20% at 12 months	Head nodding and persistent choreiform movements	At 3 months C+2-/2.4 V/150 µS/180 Hz C+10-/1.9 V/150 µS/180 Hz	At 4 years follow up, reprogramming to C+2-/2.7 V/90 µS/130 Hz C+10-/2.2 V/90 µS/130 Hz TBZ 50 mg, CBZ 600 mg, Clonazepam 0.75 mg and Baclofen 20 mg	Improvement of head nodding movements but persistence of choreiform movements and dysarthria

TS	Complex motor tics	10	11	15	5	M	Haloperidol 15 mg Risperidone 6 mg Aripiprazole 20 mg Clonidine 0.4 mg Clonazepam 4 mg Valproate 800 mg Fluoxetine 20 mg Tetrabenazine 25 mg rTMS	BL GPi DBS	Improvement in tic severity scores (YGTSS scores) by 72%	Slight worsening of motor and vocal tics (YGTSS 30/100) at 1 year with psychiatric manifestations like defiant and demanding behaviours	C+1-/3.5 V/60 µS/130 Hz C+9-/3.5 V/60 µS/130 Hz C+1-/3.5 V/60 µS/130 Hz	At 3 years follow up, Amisulpiride 300 mg and Clonazepam 1 mg and behavioural therapy	Mild improvement in behavioural symptoms


# Patient presented at 8 years of age when she only had hepatic manifestations without neurological involvement. AAO: age at onset, AAP: age at presentation, AAS: age at surgery, BFMDRS: Burke-Fahn-Marsden dystonia rating scale, BL: bilateral, CBZ: carbamazepine, DBS: deep brain stimulation, DOI: duration of illness before surgery, DYT: DYT-TOR1A, F: female, FTMRS: Fahn-Tolosa-Marin tremor rating scale, gm: gram, GPi: globus pallidus interna, M: male, mg: milligram, NA: Not applicable, NAC: Neuroacanthocytosis, NBIA: Neurodegeneration with brain iron accumulation, NPC: Niemann-Pick disease type-C, Px: pallidotomy, rTMS: repetitive Transcranial magnetic stimulation, Sx: surgery, TBZ: tetrabenazine, THP: trihexyphenidyl, TS: Tourette syndrome, Tx: thalamotomy, UL: unilateral, UHDRS: Unified Huntington disease rating scale, WD: Wilson’s disease, YGTSS: Yale global tic severity scale.

### Surgical intervention: Lesional surgeries

Eight patients (47.1%) underwent lesioning surgery after a mean duration of illness of 6.5 ± 5.2 years prior to surgery. The primary indication was tremor in four patients (57.1%), all of whom had WD (n = 4). Three patients with dystonia (37.5%), two with WD and one with NBIA underwent lesioning. A single patient with combined tremor and dystonia who underwent lesioning had NBIA as underlying etiology. Among patients with tremor, three patients underwent unilateral ventralis intermediate nucleus (ViM) thalamotomy (left-2, right-1) whereas one patient underwent bilateral ViM thalamotomy in 2 stages 6-months apart. All patients with dystonia underwent bilateral pallidotomy. The patient with tremor and dystonia (patient NBIA2) underwent left ViM thalamotomy as the tremor was more disabling (Tables 1, 2).

### Surgical outcomes of patients with dystonia

**Figure 2:**
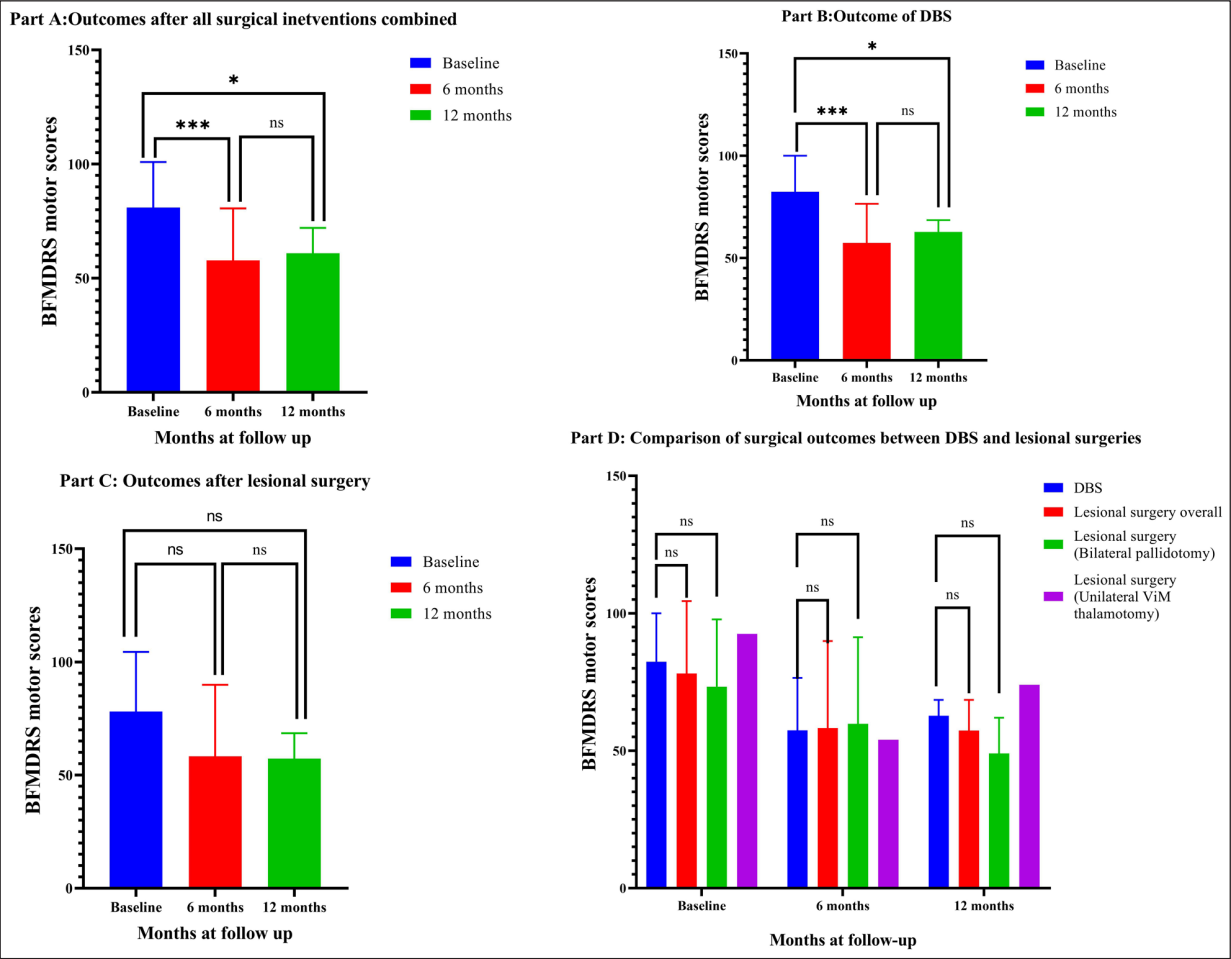
Outcome in patients with dystonia. A-Outcome after all surgical interventions combined, B-Outcome of DBS, C-Outcome after lesional surgery, D-Comparison of outcomes between DBS and lesional surgeries.

Total eleven patients had significant dystonia in our cohort of which ten patients underwent surgeries primarily for dystonia (DBS-7, pallidotomy-3). Irrespective of the type of surgical intervention, statistically significant improvement was noted in the BFMDRS motor scores at 6-months(p < 0.001) and 12-months interval (p = 0.02) compared to the baseline. Lesioning surgery led to a reduction of BFMDRS score by 26.9 ± 19.5% (p = 0.11) at 6 months and 17.4 ± 3.1% (p = 0.06) at 12-months. On the contrary, in the DBS subgroup, the score reduction was by 31.6 ± 16.2% (p = 0.001) and 24.8 ± 12.1 % (p = 0.010) at 6-months and 12-months interval, respectively. However, there was no statistically significant improvement at 12-months compared to the 6 months scores in either subgroup. When the DBS and lesioning groups were compared with each other, there was no significant difference either in the baseline (p = 0.75), or in the 6-months (p = 0.95) and 12-months follow-up scores (p = 0.53). There was no significant difference either when 6-months and 12-months outcome was compared between WD and NBIA spectrum subgroups (p = 0.18). Comparison between GPi DBS and bilateral pallidotomy showed no significant difference in mean BFMDRS motor scores at baseline (p = 0.66), 6 months (p = 0.93) or 12 months follow-up period (p = 0.48). Pairwise comparison on average BFMDRS scores, using two-way repeated measures ANOVA with Bonferroni correction, across the timepoints between GPi DBS and pallidotomy revealed a trend favouring DBS even though statistically not significant (p = 0.06) (Tables 1, 2, Figures 1 and 2).

### Surgical outcomes of patients other than dystonia

Five patients (WD-4, NBIA with tremor and dystonia-1) underwent lesioning surgery for medically refractory tremors. Objective assessment of tremor severity and functional impairment by FTM scores revealed satisfactory response at various time-points after surgery. Mean FTM scores’ reduction at last follow-up, which varied from 6 months to 16 years, was 85.0 ± 7.8%. A single patient with neuroacanthocytosis who underwent DBS for generalized chorea showed minimal improvement in UHDRS score from 36 to 33 (8.3%) at 6-months follow-up and to 30 (16.7%) at 12-months. One patient with Tourette’s syndrome showed remarkable improvement in complex motor and vocal tics by 72% on YGTSS scores at 9-month following bilateral GPi DBS. Additionally, there was reduction in HDRS scores (8/52 to 2/52), and HARS scores (7/56 to 2/56) for depression and anxiety respectively. OCD scores (YBOCSS) remained 0 at follow up. This patient was tried on several drugs for varying intervals upto four years and repetitive transcranial magnetic stimulation (rTMS) before he underwent DBS (Table 2).

### Complications of surgeries

Speech impairment was the most common adverse event observed in 7 patients, four with DBS (Patient-WD4, NBIA5, NPC and NAC) and three with bilateral pallidotomy (Patient-WD1, WD2 and NBIA1). Two patients (patient-WD1 and NBIA1) developed tongue dystonia while three (Patient-WD2, NBIA2, and NBIA5) developed blepharospasm subsequently treated with botulinum toxin therapy. One patient (Patient-WD3) developed perioral dyskinesia post-operatively. Patient-NBIA3 who had undergone bilateral GPi-DBS developed status dystonicus requiring intensive care in the immediate post-operative period and improved with standard medical management and initiation of DBS after a period of six days. Surgical site infection occurred in one patient (NBIA4, case vignette in supplementary material, Video e1) following bilateral GPi DBS necessitating removal of DBS leads. The infection subsequently subsided with no residual complications and patient continued to have sustained improvement even after lead removal. Transient imbalance was seen in two of the patients. Both these patients also had post-surgical pneumocephalus. (Tables 1, 2).

### Long term follow-up

Among WD, long term follow-up was available for three patients (WD6, 2-years; WD7, 16-years; WD8, 2-years) who had undergone unilateral thalamotomy for tremor and all three had persistent reduction in tremor and were functionally independent. Clinical details of WD8 are provided in supplementary material and Video e2. Among patients with NBIA, long term follow up was available for four. At 2 years follow up, three of the patients (NBIA2, NBIA3 and NBIA5), continued to have persistent dystonia, and required anti-dystonic medications. All three patients reported initial improvement of dystonia at 6 months which was followed by a gradual worsening. Patient NBIA-2, who also had concomitant tremors, exhibited remarkable improvement in tremors in contrast to his dystonia component. Patient-NBIA4, is discussed in case vignette (Supplementary material, Video e1). A single patient of neuro-acanthocytosis showed minimal improvement in chorea at 4 years follow-up compared to baseline. The patient with Tourette syndrome at 18 months follow-up showed mild worsening of YGTSS scores from 27 at 9 months to 30/100 with mild improvement in behavioural symptoms. The patient of NPC who underwent DBS, showed promising results with significant improvement at one year follow-up from BFMDRS motor score of 109 to 68, initially. However, at her second year of follow-up there was worsening of generalized and orolingual dystonia, to BFMDRS motor scores of 96.

### Case vignettes

#### Patient NBIA4

This 4-year-old girl presented with generalized dystonia of one year duration. The clinical presentation of pediatric dystonia, significant bulbar involvement and opisthotonus and MRI brain finding of eye-of-tiger appearance suggested a diagnosis of NBIA-pantothenate kinase-associated neurodegeneration (NBIA-PKAN). She was started on medical management which was escalated to tetrabenazine, trihexyphenidyl, clonazepam over a period of next 3 years. She responded unsatisfactorily to medical management and was subsequently taken for DBS. Her BFMDRS score at presentation was 56 which worsened to 92 pre-operatively. She made significant clinical improvement. Her BFMDRS scores improved to 64 and 59 at 6-months and 12-months follow-up respectively (Video e1). Unfortunately, during the follow-up at 2 years, she developed surgical site infection of the cranial wound which mandated removal of leads and intravenous antibiotics after which the infection was controlled. However, she continued to have beneficial effects probably due to the lesional effect. Thereafter, she was lost to follow up. Later, DBS leads were re-implanted at outside centre. Unfortunately, she passed away following chest infection.

#### Patient WD8

This 20-year-old lady, diagnosed patient of WD on treatment, presented to us with severely disabling and medically refractory tremors of 4 years duration. The patient had FTM score of 82 at presentation which did not improve significantly over next two years despite being on penicillamine, zinc, clonazepam, levodopa-carbidopa and propranolol. Subsequently, she underwent left ViM thalamotomy and had a remarkable improvement (93%) in tremors to a score of 5 at 1 year of follow-up (Video e2).

#### Patient DYT1

A13-year-old boy presented with progressive isolated generalized dystonia of 4-years duration, with the onset from right lower limb. At the time of admission, the patient was severely disabled BFMDRS motor score for dystonia of 64. MRI brain with spine was normal except for scoliosis. Genetic testing revealed a previously reported heterozygous 3-bp deletion (c.907_909del) in exon-5 of *TOR1A* gene confirming the diagnosis as DYT-TOR1A. Patient was on medical management with tetrabenazine (75 mg/day), trihexyphenidyl (12 mg/day) and levodopa 400 mg but showed unsatisfactory clinical response over the next one year. Subsequently, he underwent bilateral GPi-DBS. At 6-months follow up, patient reported significant clinical improvement with reduction in BFMDRS scores to 23. Post-surgery tetrabenazine and levodopa was stopped and was maintaining well on trihexyphenidyl 12 mg during last follow up at 9 months (Video e3).

#### Patient NBIA2

This 30-year-old gentleman, diagnosed case of NBIA, presented with features suggestive of early childhood onset generalized combined dystonia with associated tremors from the age of 13 years. Patient was severely disabled due to medical refractoriness of his symptoms. He was on trihexyphenidyl (18 mg/day) and baclofen (30 mg/day). Examination revealed BFMDRS motor scores of 92.5 and FTM score of 54. Patient underwent left ViM thalamotomy. There was notable improvement in dystonia to BFMDRS score of 54 at 6 months follow up, which however was not sustained as the score dropped off to a score of 74 at 12 months and 84 at 3 years. However, tremors showed a sustained improvement to a score of 12 at 3 years follow up. Post-operatively, patient developed blepharospasm, which subsequently improved. There was no response in his lingual dystonia, hence botulinum toxin was administered, in addition of continuation of medical therapy.

## Discussion

In this retrospective chart review, we report a series of patients with RMDs who underwent functional neurosurgery for various medically refractory disabling movement disorders. Dystonia was the most common phenomenological indication with WD being the most common aetiology. Overall, the outcome was modest in both DBS and lesioning groups and was better in the first six months compared to the one year follow up. There was no significant difference in the outcome either with respect to the surgery (DBS versus lesioning) or the aetiology (WD versus NBIA). However, patients with tremor who underwent ViM thalamotomy had excellent and sustained response over long follow-up. Speech impairment was the most common post-surgical complication and life-threatening complications were observed in two patients; post-operative status dystonicus requiring intensive care in one patient and infection of DBS lead requiring lead removal in the other.

### Outcomes based on phenomenology

Generalized dystonia is often a highly disabling syndrome and usually has unsatisfactory response to medications further limited by side effects at the high doses required for control of dystonia. Functional surgical treatment options either in the form of DBS or lesioning surgeries can up to a certain extent fill this therapeutic gap. However, the response may not be uniform and depend on various factors such as the underlying aetiology, duration of illness before surgery, nature of progression, underlying disability, and other associated neurological and systemic features. Whereas primary isolated dystonia, lower preoperative score, early surgery predicted a better outcome, combined and progressive dystonia tend to do worse with DBS [16,17]. In our report as well, among dystonia, patient with DYT-TOR1A, an isolated dystonia had the maximum improvement. The other patients with dystonia were secondary to WD, NBIA or NPC and had mild to moderate response and often were not sustained at one year follow up.

Even though effective, in some patients, DBS may not be feasible due to lack of accessibility and affordability. In such patients, bilateral pallidotomy can be an effective treatment alternative [18]. In addition, pallidotomy is devoid of hardware related adverse effects and need of long-term programming. However, bulbar adverse effects can be more in pallidotomy. In patients with affordability and accessibility issues, having contraindication to DBS, and in those in whom severity of dystonia outweighs the risk of permanent speech disorders, one may consider pallidotomy. In our cohort, three patients underwent bilateral pallidotomy for dystonia due to affordability issues and had modest response. In line with previous studies, both DBS and pallidotomy had similar overall response in our cohort. However, the sample size was too small [19].

Similarly, thalamotomy can be a good alternative to DBS in patients with medically refractory tremor. Often it is done unilaterally, opposite to the most affected side owing to the increased risk of adverse events with bilateral thalamotomy. All patients underwent ViM thalamotomy for refractory tremor in this study and all had moderate to excellent response that was sustained in the majority. Tremor of various aetiologies respond to thalamotomy with minimal side effects and should be considered in patient having predominantly unilateral tremor or in those not affordable for DBS especially so, when it is the dominant and disabling symptom irrespective of the underlying aetiology [14].

### Outcome based on aetiology

#### Wilson’s disease

Prior studies in the field investigating the impact of surgical interventions among patients of WD with medically refractory movement disorder have shown variable results. For instance, in a study on 27 patients of WD, 17 of them reported clinical improvement after stereotactic destruction of ventrolateral nucleus of thalamus and subthalamus (Table 3) [20]. However, five patients died of surgery related complications. They concluded significant improvement in hyperkinesia and greater appropriateness of unilateral surgery compared to bilateral ones [20]. Another study reported clinically significant benefit in dystonic symptoms along with improvement in functional activity following bilateral GPi stimulation [21]. Posterior subthalamic area DBS led to significant benefits in controlling refractory tremors (Fahn-Tolosa-Martin tremor rating scale, FTMTS 4/144 with neurostimulator ON and 74/144 in OFF state) in a patient of proven WD. There was significant albeit slower improvement in dystonia as well [22]. Our study which comprised 8 patients of WD, projected similar results with significant improvement in tremors in all 4 cases but much lesser benefit in dystonia.

**Table 3:** Literature review on surgical management of RMDs.


STUDY	STUDY DESIGN	SAMPLE SIZE	STUDY SUBJECT(S)	SURGERY	OUTCOMES	ADVERSE EVENT PROFILE

*Wilson’s disease*						

Starikov et al. 2000 [20]	Case series	27	16 patients with tremor form, 8 patients with tremor-rigidity form and a single patient with extrapyramidal-cortical form	Stereotaxic surgery of ViM and subthalamus	Significant decrement in hyperkinesia was reported in 17 patients	5 deaths of which 2 had bleeding in the treatment zones, 1 brainstem bleed and extension of destruction into the internal capsule and hypothalamus

Sidiropoulos et al. 2013 [21]	Case report	1	Patient of Wilson’s disease with moderate to severe dystonic symptoms	Bilateral GPi DBS	At 20 weeks of stimulation, positive impact on caregiver’s burden, especially in regard to hand dexterity, and gait	No adverse events were reported

Low et al. 2019 [22]	Case report	1	Patient of Wilson’s disease with tremors and left sided choreo-athetosis. Tremors were dominant	Posterior subthalamic DBS	In view of dominant tremor and marked atrophy of lentiform nuclei, posterior subthalamic area was chosen. Early tremor suppression was achieved. Dystonia responded after 1 year	No adverse events were reported

Neurodegeneration with brain iron accumulation						

Umemura et al.2004 [25]	Case report	1	36-year-old patient of proven PKAN with generalized dystonia for 28 years	Bilateral GPi DBS	Improvement in motor score from 112 to 22.5 out of 120 with sustained benefit at 1 year follow-up	No adverse events were reported

Castelnau et al. 2005 [23]	Case series	6	Patients with genetically confirmed PKAN	Bilateral GPi DBS	Improvement in painful dystonic spasms and motor scores. Less significant improvement in BFMDRS disability scores. DBS of the subthalamic nucleus was less beneficial in 3 patients with PKAN compared to the pallidal or thalamic stimulation	No adverse events were reported

Krause et al. 2006 [19]	Case report	1	13-year-old genetically proven patient of PKAN, intractable dystonia for 7 years	Bilateral GPi DBS	Improvement in motor scores by 70 % in 1 year. Over the next 5 years, there was gradual deterioration, but there was still functionally meaningful benefit	Fixed posturing and bradykinesia worsened

Shields et al. 2007 [26]	Case report	1	18-year-old with genetically proven patient of PKAN with dystonia for 9 years	Bilateral GPi DBS	Improvement in motor scores on BFMDRS – 86 to 66 out of 120. Patient was able to ambulate post-surgery	No adverse events reported

Mikati et al. 2008 [28]	Case report	1	11-year-old girl with early onset genetically proven PKAN	Bilateral GPi DBS	Marked improvement in motor and functional scores*. Device infection at 3 months led to its removal, following which her motor symptoms and functionality worsened.	Patient returned to pre-surgical state in 4 months

Sathe et al. 2013 [24]	Case report	1	10-year-old girl with PKAN with severe generalized dystonia, with blepharospasm and in a wheel-chair bound state	Bilateral GPi DBS	Sustained improvement in BFMDRS score for 120/120 to 42.5/120 post-operatively at 15 months follow up	No improvement in speech

*Choreacanthocytosis*						

Shin et a. 2011 [30]	Case report	1	39-year-old lady of genetically proven ChAc with refractory hyperkinetic movement and truncal bending spasm for 4 years duration	Bilateral GPi DBS	Improvement in symptoms which sustained at 13 months	No adverse events reported

Li et al. 2012 [31]	Case report	2	2 patients (39 and 30-years of age) with genetically proven ChAc of 22 and 12-years duration respectively	Bilateral GPi DBS	Chorea improved markedly 4 weeks post-stimulation. Dystonia showed only mild improvement	No adverse events reported

Ruiz et al. 2009 [32]	Case report	1	35-year-old of proven ChAc with oromandibular dystonia, dysarthria with chorea	Bilateral GPi DBS	Improvement in chorea and dystonia	Worsening of truncal spasms

Wihl et al. 2001 [34]	Case report	1	38-year-old male of proven ChAc with chorea, oromandibular dyskinesia and falls	Bilateral GPi DBS	No benefits on low or high frequency stimulation	No adverse events reported

Burbaud et al. 2002 [33]	Case report	1	35-year, lady of proven ChAc with chorea, oromandibular dyskinesia, truncal spasm	Bilateral GPi DBS	Improvement in choreic movements on high frequency stimulation (130 Hz)	No adverse events reported

*Rare inherited dystonia*						

Beaulieu-Boire et al. 2016 [17]	Prospective	11 patients with genetically proven RMD with disabling dystonia	Patients with genetically proven RMDs of 1 case each of ataxia-telangiectasia, chorea-acanthocytosis, dopa- responsive dystonia, congenital nemalinemyopathy, methylmalonic aciduria, neuronal ceroid lipofuscinosis, spinocerebellar ataxia types 2 and 3, Wilson’s disease, Woodhouse–Sakati syndrome, methylmalonic aciduria, and X trisomy	Bilateral GPi DBS ^$^	DBS effects on dystonia se verity were marginally effective, with a mean improvement of 7.9% (p = 0.39) at 1-year follow-up and 16.7% (p = 0.46) at last follow-up (mean 47.3 ± 19.9 months after surgery).	Device- related infection occurred in 1 patient (X trisomy). Two patients required a second procedure: the patient with SCA 2 received bilateral STN DBS due to lack of benefit from GPi DBS, with no further improvement, and the patient with SCA 3 had repositioning of the electrodes within the GPi, with mild benefit. Deterioration of speech was reported by 2 patients.

*Primary dystonia*						

Kupsch et al. 2006 [45]	Randomized, double-blind, sham- controlled trial	40 patients of primary generalized or segmental dystonia	40 to 75 years with disease duration of atleast 5 years, with marked disability despite optimal medical management	Bilateral GPi DBS	Improvement in mean movement score (p < 0.001). All movement symptoms showed significant improvement except speech and swallowing	Dysarthria was the most common. 22 adverse events in 19 patients.

Vidailhet et al. 2005 [39]	Prospective, controlled, multi-centre	22 patients with primary generalized dystonia	Primary generalized dystonia with a combination of segmental crural dystonia (involving one leg and the trunk) and the involvement of any other segment (the cranium, neck, or upper or lower limbs); no secondary cause, with normal neurological examination (except dystonia), MMSE score of at least 24 and normal MRI.	Bilateral GPi DBS	Significant improvement in mean motor (p < 0.001) and disability scores at 12 months (p < 0.001)	5 transient adverse events in 3 patients

Eltahawy et al. 2003 [40]	Prospective study	15 patients with primary dystonia	9 patients with primary dystonia and 6 patients with secondary dystonia (2 post encephalitis cases and 1 case each of post stroke, Huntington disease, Tardive dyskinesia and Glutaric aciduria)	Bilateral GPi DBS except for 3 cases ^@^	Primary dystonia responded better to GPi procedures (DBS and lesioning). No difference in response between pallidotomy and DBS	2/15 patients, both treated with bilateral pallidotomy, developed persistent speech impairment: hypophonia (n = 1) or dysphonia (n = 1).

Volkman et al. 2012 [44]	Randomised control trial	40 patients with primary dystonia	40 patients with severe generalised or segmental idiopathic dystonia	Bilateral GPi DBS	Significant improvements in dystonia severity at 3-years and 5-years compared with baseline were reported	Dysarthria and transient worsening of dystonia were the most common non-serious adverse events. 16/21 serious adverse events were device related.


$ One case underwent ViM Thalamotomy. # Study by Eltahawy et al. had 6 cases of secondary dystonia @ 2 unilateral lesional surgeries were done in 1 case each of post-encephalitis and post-stroke. 1 case of post-encephalitis dystonia underwent unilateral DBS * Barry-Albright dystonia scale, functional independence measures in children (WeeFIM) were used ChAc-chorea-acanthocytosis, DBS-deep brain stimulation, GPi-globus pallidus interna, MMSE-mini-mental status examination, MRI-magnetic resonance imaging, PKAN-pantothenate kinase-associated neurodegeneration, RMD-rare movement disorders, SCA-spinocerebellar ataxia, STN-subthalamic nucleus, ViM-ventrolateral intermedius nucleus of thalamus.

### NBIA

Despite limited number of studies, the surgical outcomes among patients with NBIA have been promising. In a study of 6 patients of PKAN, the largest series so far, sustained improvement in the BFMDRS motor scores was noted ranging from 46% to 91.5%. Compared to pallidal stimulation, DBS of subthalamic nucleus was found to be less beneficial [23]. Case reports prior and subsequent to this series have provided evidence on the favourable effects of surgical intervention on PKAN patients [24,25,26]. In one of the case reports, the patient with proven PKAN had gradual decline in the motor scores following initial dramatic benefits with bilateral GPi DBS. Authors attributed this to the natural course of disease in which the dystonia gradually gets converted to fixed deformities, rather than fall in DBS efficacy. The functional outcome at five years was still better compared to that of pre-operative state despite the deterioration [27]. In another report, a patient of early onset PKAN demonstrated initial significant benefit following bilateral GPi-DBS but worsened after removal of device due to infection. While the event was unfortunate, it has served to emphasise on the clinically meaningful impact of DBS in these patients [28]. De Vloo et al. in their meta-analysis on 99 patients of NBIA revealed level 4 evidence on the role of GPi DBS in improving dystonia [29]. Our current study, comprising five patients of NBIA, showed initial improvement at 6 months follow up followed by deterioration of dystonia, although severity was lesser compared to pre-surgical state, corroborating with previous studies. Patients continued to be on anti-dystonic therapy.

#### Neuroacanthocytosis

Anecdotal reports have revealed significant benefits of DBS among patients with chorea-acanthocytosis with improvements in truncal spasms, chorea and dystonia following DBS [30]. However, the rarity of the disease has precluded large scale studies. A case report on two patients with intractable chorea demonstrated definite improvements at low frequency stimulation (40 Hz) of GPi-DBS [31]. On the contrary, improvement in dystonia and chorea were exhibited on high frequency stimulation in other case reports, whereas in another report there was no clinically significant outcome [32,33,34]. Our experience with single patient revealed persistence of choreiform movements although of reduced severity at 4 year follow up.

#### Primary dystonia

Beneficial effects of DBS in cases of primary dystonia is evident based on various studies [35,36,37,38,39,40,41]. Long term experience with DBS has been satisfactory as shown in a limited number of studies till date, not only with respect to motor benefits but also in terms of disability and safety profile [42,43,44]. Significant improvement of 39–47% in the DBS group in comparison to the sham group (5–22%) was demonstrated in a 3-month randomised controlled trial of pallidal DBS in primary dystonia [45]. The current study showed remarkable benefits at 9 months follow up after bilateral GPi DBS in a single case of TOR1A mutation associated primary dystonia, re-emphasising the evidence based literature.

#### Tourette syndrome

While grey areas are still persistent with regards to the role of surgical intervention in medically refractory Tourette’s syndrome, results from a randomised cross-over trial have been promising with significant improvement in YGTSS scores post DBS [46,47]. The results from the multinational study on medically refractory tic disorder among patients with Tourette syndrome by the International Deep Brain Stimulation Database and Registry showed significant symptomatic improvement after DBS implantation (from mean YGTSS of 70.01 at baseline to 41.19 at 1-year follow-up) [48]. The common sites for implantation of electrodes were chosen as centro-median region of Thalamus followed by anterior and posterior GPi, and anterior limb of internal capsule [48]. However, the data didn’t reveal any significant difference in clinical outcomes depending on location of stimulation at 1-year follow-up. Other studies have also corroborated the positive impact of DBS on the tic severity scales amongst these patients [49]. Our experience with a single patient has shown significant initial improvement but there was relapse of motor symptoms at 1 year and mild improvement in behavioural symptoms at 3 years follow up.

### Complications of surgery

Complications of DBS can be operation, hardware or stimulation related [50]. While there were no intra-operative complications in our study, one of the patients who had undergone DBS suffered from surgical site infection which mandated surgical removal of leads. Among post-operative complications, speech impairment was the single most common complication. A recent meta-analysis showed a significant risk of speech and language related adverse effects following thalamotomy amounting to 10.3% in the unilateral dystonia subgroup which increased to 56.3% in the bilateral dystonia group [51]. The same study estimated the risk of stimulation induced dysarthria after thalamic DBS to be 24.2% with unilateral procedures and 39.2% with bilateral procedures. Status dystonicus post DBS was reported in a single patient. Although a rare complication, there are scanty reports of the same developing after an uneventful DBS surgery [52]. While sudden failure of DBS device is one of the mechanisms, it has been postulated that just like any other surgery, DBS itself can act as a trigger for worsening dystonia [53]. Blepharospasm and apraxia of eyelid opening are other known complications with DBS, which were also reported by some of our patients [54].

### Limitations

The present study has some obvious limitations such as retrospective nature, small study population, heterogenous study group, lack of a control group and non-blinded assessment. In the context of small sample size, the tests of statistical significance should be interpreted with caution. However, the rarity of such disorders and the phenotypic heterogeneity in each of them is a challenge and makes it difficult to plan a large controlled study. The present study is an attempt to report our experience and the outcome of functional neurosurgery in various rare movement disorders in order to add to the existing knowledge and to benefit the future patients.

## Conclusion

To conclude, when optimal medical therapy fails to provide a reasonable improvement in the quality of life, functional neurosurgery can be considered on a case-to-case basis. Even though the overall response is significant compared to the baseline, it is often modest and temporary. However, in patients with predominant tremor, primary dystonia, and Tourette syndrome, an excellent and lasting response can be expected and surgery should be contemplated in such cases. It is important to counsel regarding the realistic expectations of benefits and risks associated before going ahead with surgery. Management of rare movement disorders pose a significant challenge due to the rarity of the disorder, dearth of published evidence and practical limitations to perform a planned systematic trial. In this background, it is important to report such cases irrespective of the nature of the outcome to improve the overall understanding. It will help in future, in better patient selection, avoiding unnecessary invasive procedures, provide a realistic expectation and to plan a better and appropriate target. A worldwide registry of patient who have undergone functional neurosurgery for various rare movement disorder may be a right step forward in this regard.

## Abbreviation

**Table:**

BFMDRS	Burke-Fahn-Marsden-Dystonia-Rating Scale
DBS	Deep brain stimulation
ERN	European Reference Network
FTM	Fahn-Tolosa-Marin
GPi	Globus pallidus interna
HARS	Hamilton anxiety rating scale
HDRS	Hamilton depression rating scale
MRI	Magnetic resonance imaging
NBIA	Neurodegeneration with brain iron accumulation
NPC	Niemann-Pick type C
PKAN	Pantothenate kinase-associated neurode-generation
RD	Rare disease
RMDs	Rare movement disorders
RND	Rare neurologic disease
rTMS	Repetitive transcranial magnetic stimulation
S.D.	Standard deviation
UHDRS	Unified Huntington Disease Rating Scale
ViM	Ventralis intermediate nucleus
WD	Wilson’s disease
YBOCSS	Yale-Brown obsessive compulsive severity scale
YGTSS	Yale Global Tic Severity Scale
